# Identification of novel biomarker candidates by proteomic analysis of cerebrospinal fluid from patients with moyamoya disease using SELDI-TOF-MS

**DOI:** 10.1186/1471-2377-10-112

**Published:** 2010-11-08

**Authors:** Yoshio Araki, Kazuhiro Yoshikawa, Sho Okamoto, Masaki Sumitomo, Mikio Maruwaka, Toshihiko Wakabayashi

**Affiliations:** 1 Department of Neurosurgery, Nagoya University Graduate School of Medicine, 65 Tsurumai-cho, Showa-ku, Nagoya, Aichi 466-8550, Japan; 2 Promoting Center for Clinical Research, Aichi Medical University School of Medicine, Nagakute-cho, Aichi 480-1195, Japan; 3 Center for Genetic and Regenerative Medicine, Nagoya University Hospital, 65 Tsurumai-cho, Showa-ku, Nagoya, Aichi 466-8550, Japan

## Abstract

**Background:**

Moyamoya disease (MMD) is an uncommon cerebrovascular condition with unknown etiology characterized by slowly progressive stenosis or occlusion of the bilateral internal carotid arteries associated with an abnormal vascular network. MMD is a major cause of stroke, specifically in the younger population. Diagnosis is based on only radiological features as no other clinical data are available. The purpose of this study was to identify novel biomarker candidate proteins differentially expressed in the cerebrospinal fluid (CSF) of patients with MMD using proteomic analysis.

**Methods:**

For detection of biomarkers, CSF samples were obtained from 20 patients with MMD and 12 control patients. Mass spectral data were generated by surface-enhanced laser desorption/ionization time-of-flight mass spectrometry (SELDI-TOF-MS) with an anion exchange chip in three different buffer conditions. After expression difference mapping was undertaken using the obtained protein profiles, a comparative analysis was performed.

**Results:**

A statistically significant number of proteins (34) were recognized as single biomarker candidate proteins which were differentially detected in the CSF of patients with MMD, compared to the control patients (p < 0.05). All peak intensity profiles of the biomarker candidates underwent classification and regression tree (CART) analysis to produce prediction models. Two important biomarkers could successfully classify the patients with MMD and control patients.

**Conclusions:**

In this study, several novel biomarker candidate proteins differentially expressed in the CSF of patients with MMD were identified by a recently developed proteomic approach. This is a pilot study of CSF proteomics for MMD using SELDI technology. These biomarker candidates have the potential to shed light on the underlying pathogenesis of MMD.

## Background

Moyamoya disease (MMD) is characterized by progressive stenosis or occlusion of the bilateral internal carotid arteries associated with compensatory abnormal vascular network, so called moyamoya vessels [1]. A Japanese survey of 2075 patients with MMD found an annual incidence of 0.35, annual prevalence of 3.16 per 100,000 and a tendency of occurrence in the younger generation [2]. According to a world distribution analysis, a relatively large number of patients with MMD are present in East Asia, but rarely in Europe and the Americas [3]. Such regional and racial differences in susceptibility and familial occurrence in 10% of MMD cases [4] suggest that a genetic predisposition may be associated with the etiology and pathogenesis of this disease. Both 3p24-26 [5] and 8q23 [6] in genome-wide analyses, in addition to both 6q25 (D6S441) [7] and 17q25 [8] in chromosomal level analyses, have been identified in familial MMD and were recognized as possible sources of MMD. Pathologically, stenosis or occlusion of internal carotid arteries has been attributed to eccentric fibrocellular thickening of the intima following proliferation and necrosis of smooth muscle cells, which are associated with the thinning of the media [9,10]. These processes are reported to be regulated by the expression of several growth factors related to angiogenesis: transforming growth factor-β [11], basic fibroblast growth factor [12], hepatocyte growth factor [13], hypoxia inducible factor-1 [14] and vascular endothelial growth factor [15]. An increasing number of reports have been focusing on not only angiogenesis related to growth factors but vasculogenesis. Vasculogenesis is considered the pathway for adult neovascularization, which induces the formation of new blood vessels from circulating bone marrow-derived endothelial progenitor cells rather than from local endothelial cells regulated by growth factors [16,17]. It has been hypothesized that aberrant vasculogenesis contributes to vascular abnormalities including MMD [18]. Despite the establishment of approaches for clarifying the disease mechanisms of MMD, the direct underlying pathogenesis remains unclear. One approach utilizing proteomics has revealed disease-associated proteins as novel biomarkers and characterized their function in pathogenesis and development of the disease [19,20]. Among the many different types of approaches for CSF investigation [21], SELDI-TOF-MS technology [22] allows for high-throughput analysis of samples with diverse functionalization on surfaces (ProteinChip, Bio-Rad Laboratories, Hercules, CA, USA) and has been successfully used to identify protein profiles of central nervous system disorders [23,24]. The objective of this study was to apply SELDI-TOF-MS technology to identify candidate proteins in the CSF for use as biomarkers of MMD.

## Methods

### Patient Population

For proteomic analysis, CSF samples were prospectively collected from a consecutive sequence of 20 patients with MMD (11 male and 9 female; mean age, 21 years; range 1-54 years) admitted to Nagoya University Hospital, Nagoya, Japan, between February 2008 and December 2009. Diagnosis of MMD was determined by cerebral angiography or magnetic resonance imaging/angiography according to the guidelines set by the Research Committee on Moyamoya Disease (Spontaneous Occlusion of Circle of Willis) of the Ministry of Health and Welfare of Japan [25]. Type of onset of MMD includes 12 transient ischemic attacks, four cerebral infarctions, one hemorrhage and two asymptomatic cases. According to the guidelines for the diagnosis of MMD, "definite MMD" cannot have originated from any other underlying disease; the presence of an associated disease is classified as "quasi-MMD". On the other hand, for proteomic analysis of the CSF used in this study, results are thought to be unsusceptible to the underlying disease. That is, both classifications of the disease (definite or quasi) have similar clinical characteristics and treatments. Therefore, two of the patients in this study presenting with Down syndrome were categorized as MMD (Table 1). As controls, 12 patients were recruited from among those admitted to Nagoya University Hospital for surgical treatment of other clinical conditions, including nine cases of cerebral aneurysm. The control patients were matched according to gender; however, their overall age was older than that of patients with MMD, as patients with MMD tend to be younger than those with cerebral aneurysm (75% of control patients), which mainly affects middle-aged individuals.

**Table 1:** Clinical characteristics of each patient group in this study

		No. of patients (%)		
		
		MMD	Controls	***P *****value**
	Variables	n = 20	n = 12	
Age	Mean ± SD, years	20.5 ± 17.4	61.2 ± 9.1	<0.01
Sex, n (%)				
	Male	11 (55.0)	3 (25.0)	0.1
	Female	9 (45.0)	9 (75.0)	
Type of onset, n (%)				NA
	TIA	12 (60.0)		
	Infarction	4 (20.0)	2 (16.7)	
	Hemorrhage	1 (5.0)		
	Asymptomatic	2 (10.0)	7 (58.3)	
	Others	1 (5.0)	3 (25.0)	
Associated disease, n (%)				NA
	None	18 (90.0)		
	Down syndrome	2 (10.0)		
	Cerebral aneurysm		9 (75.0)	
	Others		3 (25.0)	
Suzuki's angiographycal stage (1-6)	Mean ± SD	3.05 ± 0.9		NA

TIA, transient ischemic attack; MMD, moyamoya disease; SD, standard deviation.

### Sample Collection

All CSF samples were collected after obtaining informed written consent from the patients following approval from the Nagoya University School of Medicine Ethical Review Board. CSF was obtained for all subjects in the same manner during the first phase of the operative procedure after the arachnoid membrane was dissected. Blood contamination was avoided as much as possible by carefully performing hemostasis. CSF sampling was performed at least 4 weeks after the stroke event associated with MMD or other pathogenesis. Within 2 h of collection, the CSF sample was stored in 1.5-ml cryo-tubes, centrifuged at 3000 rpm for 10 min at 4°C, and the supernatants were frozen and stored in approximately 1-ml aliquots at -80°C until analysis.

### Sample preparation and analysis

#### CSF preparation

The CSF samples in each cryo-tube were thawed in water at room temperature, then kept on ice and well agitated before subjected to ProteinChip array. The samples were diluted by each binding/washing buffer (0.2 M citrate-phosphate, pH 5 or 7, or 0.2 M Tris-HCl, pH 9) at a final ratio of 1/5.

#### ProteinChip array pretreatment

Q10 (strong anion exchanger) ProteinChip array (Bio-Rad Laboratories) was used for protein profile analysis. The chips were equilibrated prior to the CSF sample addition with binding/washing buffer for 2 × 5 min.

#### ProteinChip array preparation

Diluted CSF (20 μl) was applied on a spot in the chip and incubated for 40 min in a humid box at room temperature. After allowing the sample to bind, the remaining sample was removed from all spots by immediately washing the array with the corresponding binding/washing buffer 3 × 5 min. The arrays were rinsed with 150 μl distilled water twice and allowed to air-dry for 15 min. Then, 1 μl saturated energy absorbing molecule (EAM) solution (sinapinic acid in 50% acetonitrile and 0.5% trifluoroacetic acid) was applied to each spot and again allowed to air-dry before analysis by the ProteinChip reader.

### SELDI-TOF-MS and data acquisition

#### Mass spectral processing

The protein mass spectral data was generated with ProteinChip System 4000 SELDI-TOF mass spectrometer (Enterprise version; Bio-Rad Laboratories) using automated data collection protocol with Ciphergen Express version 3.0.6 software interface (Bio-Rad Laboratories). The optimal laser intensity range for spectra generation was predetermined to be 2800 to 3500 nJ by manual laser shots. For determining the maximum protein peak yield and spectral reproducibility, data were collected between 0 and 100 kDa with the ion focus mass set to 7000 Da and the matrix attenuation to 1000 Da. After two initial warming shots, 53 points equally distributed over the chip spot surface were lased 10 times for ionization of the proteins.

#### Peak detection of SELDI protein profiles

All the obtained spectra were internally mass-calibrated and normalized to the total ion current of an m/z value more than 1000 for avoiding the signal interference from EAM. Expression difference mapping (EDM) was performed automatically using Ciphergen Express data management software version 3.0.6 under the following conditions: signal/noise ratio of 5 or higher for the first pass, 2 for the second pass, and presentation in at least 20% of spectra for identification.

#### Data analysis for single biomarker candidate protein identification

A comparative analysis of protein profiles of the MMD and control groups using univariate analysis was performed for single biomarker candidate protein identification under each pH condition. P values were calculated based on the Mann-Whitney *U *test for non-parametric data or two-tailed *t*-test for parametric data, and the area under the receiver operation characteristic (ROC) curve (AUC) of each peak cluster was determined by Ciphergen Express data management software version 3.0. A P value of less than 0.05 was considered statistically significant.

#### Data mining using classification and regression trees (CART)

Generally, stability and accuracy of the prediction model were tested to assign a training set and test set from the data set. However, as the size of the data set in this study was too small to construct an independent validation set, a tree-based model was adopted to make a prediction model for multi-biomarker candidate protein identification. This model applies non-linear regression analysis and has recently been used broadly for data mining. CART for data mining was applied as described previously [26,27]. A decision tree was generated using entropy and the Gini index for calculating a node [28]. Branching of the tree is created based on the values (node) calculated by these indices and pruned using the complexity parameter. In this study, the complexity parameter was set to 0.01. This analysis was performed with the R software environment for statistical computing (R Development Core Team, Vienna, Austria).

#### Peak reproducibility

Reproducibility of peak intensity and mass accuracy were evaluated using pooled CSF samples from two individuals from the control group. The coefficient of variance (CV) was calculated using randomly selected multiple protein peaks over the experiment as previously described [29,30]. For the assessment of peak reproducibility of the SELDI profiles, both the intra-assay (spot-to-spot) and inter-assay (chip-to-chip) CV were determined.

## Results

### Peak reproducibility

In this study, the intra-assay (spot-to-spot) CV was 17.38% for peak intensity and 0.11% for mass accuracy. The inter-assay (chip-to-chip) CV was 25.11% for peak intensity and 0.14% for mass accuracy, indicating acceptable reproducibility of the spectra.

### SELDI-TOS-MS protein profiling and data analysis

#### SELDI-TOF-MS analysis for CSF samples

After making the EDM cluster, the CSF samples were analyzed in a binding/washing buffer with three different pH conditions (pH 5, 7 and 9). Between 1 to 100 kDa m/z, 54, 40 and 59 m/z peak clusters were generated for the pH 5, 7 and 9 conditions, respectively; the respective minimum peaks were 1027.73, 1041.82 and 1026.39, and the respective maximum peaks were 93797.02, 94648.40 and 95278.35.

#### Univariate analysis using EDM data for identification of single biomarker candidate proteins

**Figure 1:**
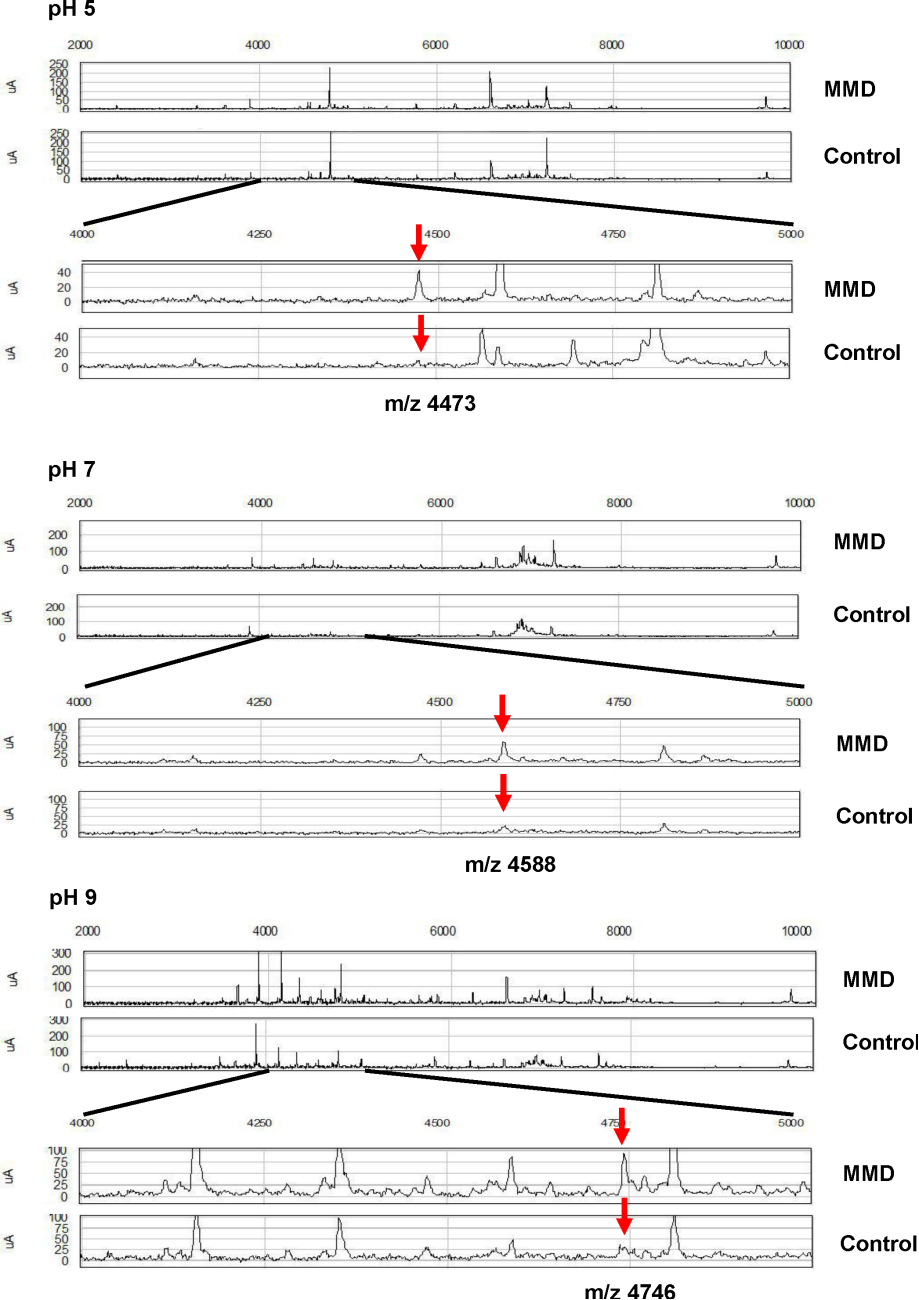
**Mass spectra of representative single biomarker candidate proteins in CSF under different pH conditions. **Protein profiles of the MMD and control groups were generated using Q10 (strong anion exchanger) array. For each pH condition, the upper two spectra are protein profiles obtained between m/z 2,000 and 10,000, and the lower two spectra are expansions showing the peak intensities around m/z 4473, 4588 and 4476 for pH 5, 7 and 9, respectively. All representative peaks (red arrows) are larger for the MMD than control group under each pH condition, as determined by SELDI-TOF-MS.

**Figure 2:**
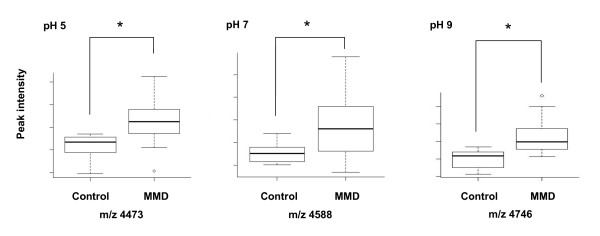
**Peak intensity of representative single biomarker candidate proteins in the MMD and control groups. **Peak intensities of representative single biomarker candidate proteins detected using SELDI-TOF-MS are significantly larger in the MMD than control group under each pH condition (m/z 4473, 4588 and 4476 for pH 5, 7 and 9, respectively). The box-whisker plots indicate the median value (*thick line*) and the 25th (*lower line of box*) and 75th (*upper line of box*) percentile; T bars indicate the 10th and 90th percentile. The p values between the groups were calculated using Mann-Whitney *U *test. *P < 0.01.

For identification of single biomarker candidates in each pH condition, univariate analysis was performed using EDM data. Table 2 shows 34 biomarker candidate proteins differentially expressed among the MMD and control groups (P < 0.05); m/z was less than 10 kDa and ranged from 1041.03 to 50989.55, for which 15 proteins were significant (P < 0.01). Of the 34 biomarker candidates, 15, 5 and 4 were detected under pH 5, 7 and 9 conditions, respectively. The mean ROC AUC value of each cluster was 0.7723, ranging from 0.7 to 0.9. Among these protein candidates, 19 were up-regulated and 15 were down-regulated in the MMD group compared to the control group. There were 11 peak clusters thought to be originated from 5 m/z peak clusters; m/z 4473.46 at pH 5 and 4475.10 at pH 7, m/z 4589.34 at pH 9, 4588.73 at pH 5 and 4588.87 at pH 7, m/z 6941.48 at pH 9 and 6942.96 at pH 7, m/z 13882.19 at pH 9 and 13877.49 at pH 7. Within these clusters, there is a minimal difference in m/z (<5.3). Spectra of representative single biomarker candidates under each pH condition using SELDI ProteinChip analysis are shown in Figure 1 for each CSF sample from the MMD and control groups. All proteins were up-regulated in the MMD group. A box-whisker plot of the peak intensities for representative single biomarker candidate proteins in each group is demonstrated in Figure 2. For the MMD group, the mean peak intensity of m/z 4473 at pH 5 was 22.64 ± 9.72 (mean ± standard deviation), that of m/z 4588 at pH 7 was 56.67 ± 28.42, and that of m/z 4746 at pH 9 was 44.94 ± 18.76; those of the control group were 11.78 ± 5.16 30.62 ± 8.42, and 20.37 ± 10.24, respectively. All peak intensities of each cluster were significantly larger in the MMD than control group (P < 0.01).

**Table 2:** Single biomarker candidates from univariate analysis of CSF sample comparison of MMD and control groups

***P***-value*	m/z	pH of binding/washing buffer	ROC AUC	Level in MMD
1.60E-04	4746.76	pH 9	0.9000	up
5.32E-04	4566.71	pH 5	0.8583	down
6.14E-04	4473.46 ^a^	pH 5	0.8750	up
1.08E-03	4157.65	pH 9	0.8250	up
1.41E-03	4589.34 ^b^	pH 9	0.8250	up
1.41E-03	50989.55	pH 9	0.8417	down
1.41E-03	4588.73 ^b^	pH 5	0.8250	up
3.09E-03	4588.87 ^b^	pH 7	0.8000	up
3.09E-03	6941.48 ^d^	pH 9	0.8083	down
3.51E-03	6942.96 ^d^	pH 7	0.8167	down
7.24E-03	9656.51	pH 5	0.8000	up
7.24E-03	13754.72	pH 5	0.7750	up
8.12E-03	4809.94 ^c^	pH 5	0.7667	down
9.11E-03	2406.55	pH 5	0.7667	down
9.11E-03	13882.19 ^e^	pH 9	0.7583	down
1.02E-02	9728.65	pH 5	0.7750	up
1.14E-02	6228.21	pH 5	0.7333	down
1.27E-02	13877.49 ^e^	pH 7	0.7917	down
1.42E-02	21299.65	pH 5	0.7583	down
1.58E-02	5781.11	pH 5	0.7500	up
1.76E-02	14060.91	pH 9	0.7417	down
1.76E-02	4695.40	pH 5	0.7417	down
1.95E-02	6240.88	pH 9	0.7500	up
2.16E-02	28095.59	pH 9	0.7583	down
2.40E-02	4475.1 ^a^	pH 7	0.7500	up
2.40E-02	7250.89	pH 7	0.7250	up
2.40E-02	3516.49	pH 9	0.7250	up
2.93E-02	6888.58	pH 5	0.7250	up
3.23E-02	3681.83	pH 9	0.7250	up
3.56E-02	1041.03	pH 9	0.7000	up
3.91E-02	7146.84	pH 5	0.7333	down
4.30E-02	4811.33 ^c^	pH 9	0.7000	up
4.71E-02	1061.94	pH 9	0.7250	up
4.71E-02	34344.75	pH 5	0.7083	down

^a-e^Values with the same lower-case letter indicate clusters most likely derived from the same proteins. *P < 0.05, Mann-Whitney *U *test

#### Data mining using CART analysis

**Figure 3:**
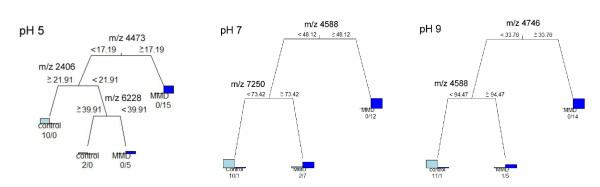
**CART analysis using peaks obtained by SELDI-TOF-MS to discriminate between patients with MMD and control patients. **The decision tree was constructed using CSF samples from 32 patients with MMD and control patients. The classification is determined starting at the roof node, following by appropriate splitting decisions based on the peak intensity at each node. If the peak intensity is lower than the cutoff intensity value, the left node is selected. This splitting process is continued until no further classification is achieved and terminal nodes are produced. Using m/z 4473, 2406 and 6338 peaks (pH 5), m/z 4588 and 7250 peaks (pH 7), and m/z 4746 and 1044 peaks (pH 9), CART for Q10 ProteinChip was applied to identify patients with MMD and control patients. The analysis correctly classified all 20 patients with MMD under pH 5 condition and 19 of 20 under the pH 7 and 9 conditions; all 12 control patients were classified under all pH conditions.

**Figure 4:**
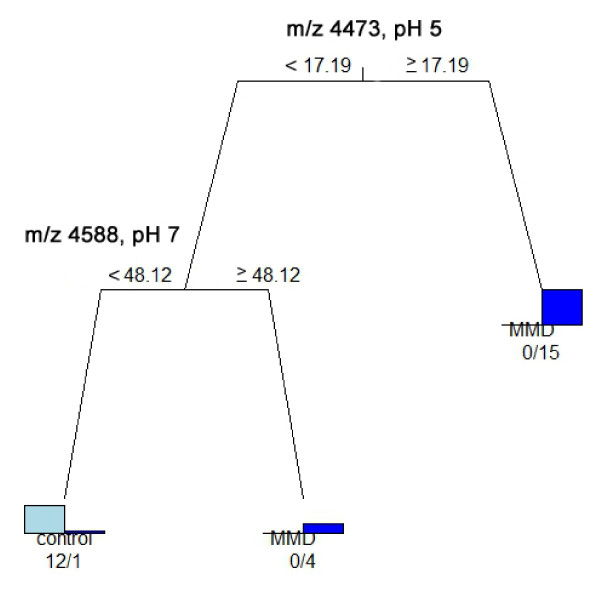
**CART analyses using all 34 single biomarker candidate proteins. **CART was analyzed for 34 single biomarker candidate proteins identified in the CSF under each pH condition (pH 5, 7 and 9) to discriminate patients with MMD from control patients. This analysis correctly classified 19 of 20 patients with MMD and all 12 control patients based on the peak intensities of the m/z 4473 peak (pH 5) and m/z 4588 peak (pH 7).

CART analysis was undergone to discriminate patients with MMD from control patients using a single biomarker obtained by SELDI-TOF-MS under different pH conditions (pH 5, 7 and 9) (Figure 3). The analysis correctly classified all 20 patients with MMD at pH 5 and 19 of them at pH 7 and 9; all control patients were classified correctly under all pH conditions. As shown in Figure 4, CART analysis was able to classify 19 of the 20 patients with MMD and all 12 of the control patients.

## Discussion

### Purpose of this study

Diagnosis of MMD is currently only achievable by radiological features using cerebral angiography or magnetic resonance imaging/angiography. However, characteristics of MMD-mimicking vascular conditions are frequently encountered on radiological imaging in the clinical setting [31,32], sometimes rendering indeterminate diagnosis of MMD. In this pilot study, we utilized novel biomarker candidate proteins identified in CSF samples by SELDI-TOF-MS to aid in the definite diagnosis of MMD. Based on our results, a mechanism for these biomarkers in association with the pathogenesis of MMD could be elucidated.

### Study design

Many factors have limited the advancement of basic research on MMD. Some of these include low mortality rate, difficulty of obtaining surgical specimens from the internal carotid artery or related intracranial vessels, and lack of an appropriate animal model [33]. Recent investigations of MMD have focused on its epidemiology [2-4], pathology [9,10], relationship with endothelial progenitor cells [16,17] and genetics [5-8], identifying some potential mechanisms and development pathways of this condition. However, their findings constitute only part of the underlying cause of MMD. As the CSF contacts the extracellular space of the brain, it contains an obvious source of biomarkers which should reflect central neuro-pathologic impairments of the brain. With the development of global proteomic research for biomarker discovery [34], previous studies have proposed many biomarkers, especially for neuro-degenerative diseases, although mainly using two-dimensional polyacrylamide electrophoresis [35,36]. A variety of recent proteomic techniques based on MS have also been utilized, including electrospray ionization tandem MS, matrix-assisted laser desorption/ionization MS and SELDI MS, with mechanically different methods of ion separation, mass accuracy and resolution, along with different pretreatments of low abundant proteins, such as found in the CSF [37,38]. Our study focused on proteomic analysis using SELDI-TOF-MS, a high-throughput, reliable technique which allows large numbers of patient samples to be investigated with a low sample volume scale, simple maneuvering and comparatively shorter time than other methods. SELDI technology has been used for a number of clinical situations and sample types to identify biomarker protein expression patterns which discriminate patients with a certain disease from control individuals [39].

We tested Q10 (strong anion exchanger) ProteinChip array to capture a wide variety of proteins derived from the CSF, including physiologically active substances ionized under physiological pH (7.2 - 7.4). We also tested CM10 (weak cation exchanger) ProteinChip array, which can indiscriminately detect many kinds of biomarker proteins, although no meaningful results were obtained using our methodology (data not shown). Q10 ProteinChip array was therefore applied in this study using a binding/washing buffer at pH 5, 7, and 9, around physiological pH conditions. This is the first study to identify biomarker candidate proteins by SELDI-TOF-MS using the CSF of patients with MMD.

### Evaluation of the results in this study

#### Definite diagnostic tool for discrimination of patients with MMD and control patients

In the course of our study using SELDI-TOF-MS, 34 candidates of single biomarker proteins within the range of 1-50 kDa were generated with Q10 ProteinChip array. SELDI technology can feasibly resolve these low molecular weight proteins which cannot be resolved by other current proteomic methods like two-dimensional electrophoresis. As a definite diagnostic tool of MMD, analysis of these proteins could adequately discriminate between the MMD and control groups (P < 0.05 and ROC AUC 0.7 - 0.9). CART analysis enabled this discrimination to provide a prediction model with relatively higher sensitivity and specificity, although the study population was too small to construct independent training and test set subjects. Therefore, a larger study population is needed for validation of these results.

#### Comparison analysis using over-expressed/down-regulated proteins in the CSF of patients with MMD

Previous studies have suggested several growth factors that are related to angiogenesis. These include transforming growth factor-β [11], basic fibroblast growth factor [12], hepatocyte growth factor [13], hypoxia inducible factor-1 [14] and vascular endothelial growth factor [15], with m/z 83133, 55960, 42005, 92670, 38200 Da, respectively. As these angiogenic factors have relatively larger molecular weights (>10 kDa), they are more accessible to purification and exact identification using conventional methodologies. For a further understanding of the underlying pathophysiology of MMD, the most significant m/z peaks were elucidated on the proteome-wide database level. Candidate protein (peptide) biomarkers were inferred using the TagIdent tool (http://au.expasy.org/tools/tagident.html) from the UniProt Knowledgebase (Swiss-Prot and TrEMBL) databases based on the definite molecular mass; the retrieval conditions were set as pI 1.0 to 14.0 and m/z ± 0.5%. Six proteins (peptides) had corresponding molecular weights of two candidates, m/z 4473 and 4588, obtained by CART analysis from the 34 single biomarker candidate proteins (Table 3). Exact identification of biomarker proteins is undoubtedly advantageous, although as in this study, purification is difficult to achieve with a lack of abundant samples from clinical subjects. Moreover, protein identification requires specialized techniques, and a highly sensitive method is needed to properly identify the target protein, especially those with a low molecular weight.

**Table 3:** Biomarker candidate proteins (peptides) in the CSF of patients with MMD

Theoretical MW (Da)	Inferred candidate protein (peptide)
4449.89	Oxyntomodulin (P01275)
4451.26	Urocortin-2 (Q96RP3)
4461.22	Beta-defensin 133 (Q30KQ1)
4493.32	Antibacterial protein LL-37 (P49913)
4585.34	Liver-expressed antimicrobial peptide 2 (Q969E1)
4586.6	Proenkephalin-A (143-183) (P01210)

The number in parentheses is the serial number of the protein in the UniProt Knowledgebase (Swiss-Prot and TrEMBL) databases.

## Conclusions

Several novel biomarker candidate proteins differentially expressed in patients with MMD and control patients were identified using a recently developed proteomic approach. This is a pilot study for CSF proteomics of MMD using SELDI technology. Multi-biomarker analysis using CART made the prediction models more specific and exhibited two m/z panels as a discriminative tool for the definite diagnosis of MMD. For further understanding of the pathogenesis of MMD, a larger number of CSF samples for validation of the analysis and well-defined methodology and techniques for exact identification of biomarkers are essential.

## Competing interests

The authors declare that they have no competing interests.

## Authors' contributions

YA and KY carried out SELDI and participated in the subject evaluations. YA, SO, SM and MM contributed to collecting CSF samples. YA, KY, SO and TW participated in study design and coordination and drafting the manuscript. All authors read and approved the final manuscript.

## Pre-publication history

The pre-publication history for this paper can be accessed here:

http://www.biomedcentral.com/1471-2377/10/112/prepub
